# Synaptic-like vesicles and candidate transduction channels in mechanosensory terminals

**DOI:** 10.1111/joa.12337

**Published:** 2015-07-14

**Authors:** Guy S Bewick

**Affiliations:** School of Medical Sciences, Institute of Medical Sciences, University of AberdeenAberdeen, UK

**Keywords:** baroreceptors, glutamate, lanceolate endings, mechanosensory terminal, metabotropic glutamate receptor, muscle spindle, synaptic-like vesicles

## Abstract

This article summarises progress to date over an exciting and very enjoyable first 15 years of collaboration with Bob Banks. Our collaboration began when I contacted him with (to me) an unexpected observation that a dye used to mark recycling synaptic vesicle membrane at efferent terminals also labelled muscle spindle afferent terminals. This observation led to the re-discovery of a system of small clear vesicles present in all vertebrate primary mechanosensory nerve terminals. These synaptic-like vesicles (SLVs) have been, and continue to be, the major focus of our work. This article describes our characterisation of the properties and functional significance of these SLVs, combining our complementary skills: Bob’s technical expertise and encyclopaedic knowledge of mechanosensation with my experience of synaptic vesicles and the development of the styryl pyridinium dyes, of which the most widely used is FM1-43. On the way we have found that SLVs seem to be part of a constitutive glutamate secretory system necessary to maintain the stretch-sensitivity of spindle endings. The glutamate activates a highly unusual glutamate receptor linked to phospholipase D activation, which we have termed the PLD-mGluR. It has a totally distinct pharmacology first described in the hippocampus nearly 20 years ago but, like the SLVs that were first described over 50 years ago, has since been little researched. Yet, our evidence and literature searches suggest this glutamate/SLV/PLD-mGluR system is a ubiquitous feature of mechanosensory endings and, at least for spindles, is essential for maintaining mechanosensory function. This article summarises how this system integrates with the classical model of mechanosensitive channels in spindles and other mechanosensory nerve terminals, including hair follicle afferents and baroreceptors controlling blood pressure. Finally, in this time when there is an imperative to show translational relevance, I describe how this fascinating system might actually be a useful therapeutic drug target for clinical conditions such as hypertension and muscle spasticity. This has been a fascinating 15-year journey in collaboration with Bob who, as well as having an astute scientific mind, is also a great enthusiast, motivator and friend. I hope this exciting and enjoyable journey will continue well into the future.

vesicles, though characteristic of (presynaptic) terminals, are certainly not restricted to them.

Bernard Katz, *Nerve, Muscle and Synapse*, p. 98.

## Introduction

**Fig 1:**
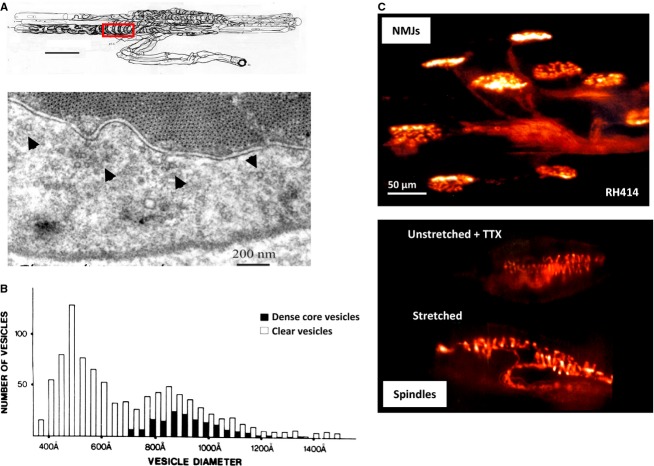
Synaptic-like vesicles (SLVs) in muscle spindle annulospiral endings. (A) The upper drawing is a reconstruction of a serially sectioned cat muscle spindle showing the incoming myelinated afferent axon arriving from below, as it then branches and eventually loses its myelin sheath to deliver a series of characteristically annulospiral endings wrapping around intrafusal muscle fibres. Scale bar: 100 μm. The red box delineates an area of terminal typically sampled to reveal the clusters of 50-nm-diameter, clear ‘synaptic-like’ vesicles within. Shown below is one such section. The regular array of contractile proteins is seen at the top, with the paler, floccular sensory nerve terminal seen below. The most obvious SLV clusters are indicated with arrowheads, but closer inspection shows that SLVs are scattered throughout. Note that the clusters are not all focussed towards the muscle fibre, i.e. they do not appear to be truly ‘synaptic’. SLVs are as likely to be clustered adjacent to terminal membrane facing away from the muscle fibre (e.g. cluster indicated by the right-most arrowhead) as towards it. (B) An historical quantification (for younger readers: 1 Å = 10^−10^ m, i.e. 10 Å = 1 nm) of the diameters of all vesicles within primary sensory endings revealed a range of diameters and a mix of clear and dense-cored vesicles. However, by far the most abundant population is about 500 Å, or 50 nm. (C) Top: fluorescent labelling of motor nerve terminals stimulated in RH414, a prototype styryl pyridinium dye used in the development of the more commonly used dye, FM1-43. During this work with Bill Betz and Steve Fadul (University of Colorado Health Sciences Center, Denver), we showed dye internalisation occurred by endocytosis with recaptured vesicle membrane. This is when we first noticed (Bottom) the characteristic labelling of the annulospiral endings of muscle spindle primary afferent terminals in the same muscle (rat lumbrical muscle). Spindle labelling occurred even if the muscle was unloaded (i.e. not stretched) and in the presence of tetrodotoxin (TTX) to block afferent discharge. Thus, electrical and mechanical activity were not required to get labelling, suggesting at least a basal level of SLV endocytosis occurs at rest. From Bewick et al. (2005) with permission.

My first contact with Bob Banks was by telephone in 1999. The call was to ask him, ‘Do muscle spindle afferent terminals contain vesicles?’ I had asked Clarke Slater (Newcastle University), who was my last postdoctoral employer, but he did not know (a very rare circumstance in itself). However, he said, ‘if anyone knows it will be Bob Banks in Durham’. And he was correct. Bob immediately replied, that, yes, 50-nm clear vesicles had been reported > 40 years earlier in mechanosensory afferent terminals at the same time as synaptic vesicles but, as they had no obvious function, this had largely been ignored ever since. He then emailed me a beautiful electron microscope image of vesicles in a terminal, plus some quantitative analysis of vesicle abundance from David Barker’s work (Fig.1A,B). Finally, Bob pointed me to p. 98 of Bernard Katz’ seminal work *Nerve, Muscle and Synapse* (Katz, 1966) and the above quotation, which reads in full,

Attempts have been made to [] classify all neuronal structures which contain vesicles as ‘chemically transmitting presynaptic terminals’. Without more direct evidence, this would be difficult to defend because the presence of vesicles, though characteristic of such terminals, is certainly not restricted to them.

And thus began the rich seam of research we continue to mine to this day.

My question was prompted by my work 10 years earlier in Bill Betz’ lab (University of Colorado), with his wonderful technician Steve Fadul, while developing the styryl pyridinium dyes (RH414, FM1-43 and FM4-64) to label synaptic vesicles in efferent terminals. While studying the activity-dependent labelling of presynaptic motor terminals with these dyes and finding it was due to synaptic vesicle recycling, we noted that they also labelled annulospiral afferent endings of muscle spindles (Betz et al. 1992). Not being spindle experts, we passed this observation on to Cuy Hunt, who immediately confirmed it. However, Cuy did not then pursue it further, so any implications of this discovery lay essentially dormant until that phone call to Durham.

As a synaptic neuroscientist, the juxtaposition of these observations – labelling, vesicles and the lack of obvious function – piqued my interest greatly. This review will describe the characterisation of the labelling mechanism, the subsequent discovery of an apparently ubiquitous glutamate secretory system for primary mechanosensory endings, the crucial role of an atypical glutamate receptor, and the potential relevance of this system to blood pressure control.

## Early studies: the classical model of mechanosensation is incomplete

**Fig 2:**
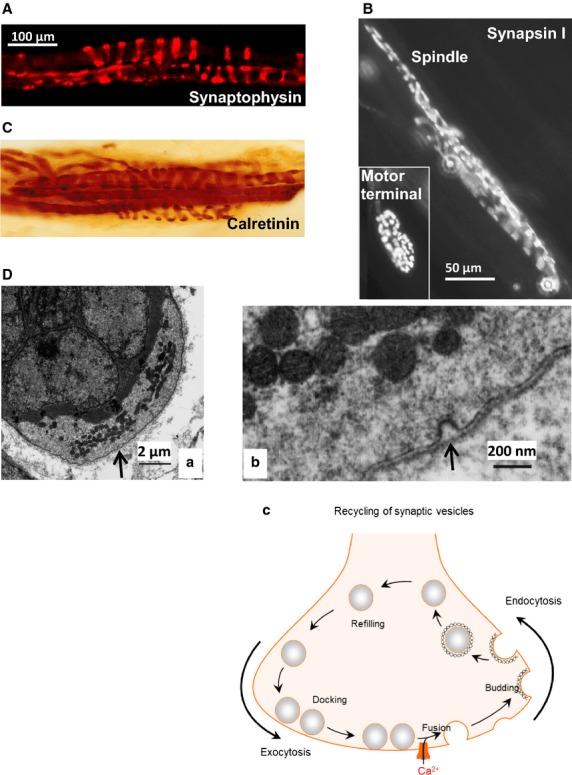
Muscle spindle primary afferents express synaptic vesicle-associated proteins and exhibit endocytosis. Immunoreactivity in muscle spindles for the ubiquitous synaptic vesicle proteins (A) synaptophysin (cat) (B) synapsin I (rat: courtesy of Arild Njå, University of Oslo) and Ca^2^^+^ -binding protein (C) calretinin (cat). Note the insert showing labelling for synapsin I in a motor nerve terminal of the same muscle in (B). (D) Evidence of endocytosis in an annulospiral terminal from a cat muscle spindle. (a) Transverse section showing the underlying nuclear bag intrafusal fibres (dark area, upper left), partially enclosed by the sensory terminal (lighter area, lower right). The arrow indicates the area of interest shown at higher magnification in (b). There are several things to note at this point. First, note the presence of a coated pit (arrow) typical of clathrin-mediated endocytosis during membrane recovery of synaptic vesicles. This is typical of membrane recovery, rather than exocytosis. Second, the pit is of approximately 50 nm diameter. Finally, the membrane recovery is occurring on the side of the terminal away from the muscle fibre, i.e. such membrane recovery can occur all over the surface of the terminal. (C) Simplified schema of vesicle recycling from exocytosis (vesicle fusion and neurochemical release), through endocytosis, via specialised budding proteins, and subsequent refilling with neurotransmitter/modulator, then docking ready for re-release. (B–D) From Bewick & Banks (2015), with permission.

The classical model of sensory neurone mechanotransduction is that surface membrane lengthening opens a stretch-sensitive Na^+^ channel, depolarising the ending, to produce the receptor potential (RP). Passive electrotonic spread of the RP to an initiation site triggers an action potential (AP) afferent discharge with a rate proportional to the depolarisation delivered. Viewed simplistically, this model relies only on Na^+^ channels (mechanosensitive for the RP, then voltage-gated for the upstroke of the AP) and K^+^ channels (repolarisation of the AP), and has no requirement for the involvement of vesicles. Having ‘re-discovered’ these synaptic-like vesicles (SLVs), and being a synaptic physiologist, the first question we had was how much further the similarities between vesicles in mechanosensory and efferent motor endings extended. A literature trawl, plus some of Bob’s own unpublished work, revealed many more similarities in anatomy and protein expression. First, all electronmicrograph (EM) studies of mammalian primary mechanosensory endings reported (or, our examination of the published EM in these studies revealed) 50-nm clear vesicles (Krauhs, 1979; Akoev et al. 1988; Zelena, 1994), with the first overt reference to their resemblance to synaptic vesicles noted in 1966 (Cauna, 1966). Second, spindle afferent immunocytochemistry revealed many synaptic vesicle-associated proteins, including synaptophysin (a ubiquitous synaptic vesicle protein) and synapsin I (Fig.2A,B; a vesicle clustering protein) – although not synapsin II (De Camilli et al. 1988). Third, a large number of Ca^2^^+^ -binding proteins are present in annulospiral endings, including calretinin (Fig.2C), calbindin D-28k, neurocalcin, NAP-22 and frequenin (Hietanen-Peltola et al. 1992; Duc et al. 1993; El-Tarhouni & Banks, 1995; Iino et al. 1998; Werle et al. 2000). Finally, elements of both the vesicle and terminal membrane SNARE vesicle docking and fusion complexes had been shown to be present, i.e. the v-SNARE synaptobrevin I/II (Li et al. 1996) and t-SNARE syntaxin IB, but not syntaxin IA (Aguado et al. 1999). SNAREs are a protein family, whose acronym derives from SNAP Receptors, which are intimately involved in rapid and specific synaptic vesicle docking and fusion events. Given the exclusively Na^+^- and K^+^-channel-dependent nature of the classical model, these findings are surprising. However, we were excited to see that they are suggestive of Ca^2^^+^ -dependent synaptic/secretory vesicle turnover.

The next question was, is there evidence that SLVs can undergo exo-/endocytosis and that this is functionally important? Bob’s image library of Ω-profiles and coated pits was clear evidence of SLV recycling (Fig.2D), while synaptic neurotoxin studies revealed its functional implications. Black widow spider venom, containing latrotoxin, elicits uncontrolled synaptic vesicle exocytosis (Ushkaryov et al. 2008). It also causes depletion of SLVs and subsequent destruction of annulospiral endings (Queiroz & Duchen, 1982), indicating the presence and functional importance of the ‘presynaptic’ proteins latrophilin and/or neurexin. A critical functional role for SLV exocytosis was first suggested by studies with tetanus toxin (Mizote & Takano, 1985). This toxin very selectively cleaves the v-SNARE synaptobrevin. When injected into cat gastrocnemius, as expected, the toxin gradually blocked nerve-stimulated muscle contraction, reflecting the progressive inhibition of neuromuscular transmitter release. Strikingly, however, it also gradually abolished muscle spindle stretch-evoked firing – and over the same time-course. Thus, an intact SNARE complex seems essential to sustain the annulospiral ending’s ability to respond to stretch. An important role for Ca^2+^ was first hinted at when Hunt et al. (1978) revealed it made a small contribution to the stretch-activated RP. A central role for Ca^2+^ in the mechanosensory responsiveness, as implied by the abundance of Ca^2^^+^ -binding proteins, was revealed when spindle responses were abolished either by removal of Ca^2+^ from the external medium, or addition of cationic Ca^2+^channel blockers (Co^2+^ or Ni^2+^/Cd^2+^; Kruse & Poppele, 1991). These observations are important for two reasons. Firstly, they show strong functional parallels with synaptic terminals: viz. an absolute requirement for Ca^2+^, perhaps to support SLV recycling; and, secondly, and perhaps most tellingly, they show the classical model is incomplete. This model being entirely based on monovalent cations (Na^+^ and K^+^), it has no requirement for Ca^2+^ and, therefore, there is no reason why Ca^2+^ should have such a profound effect.

Thus, the literature held many clues that the classical model was incomplete. That these clues lay largely ignored was not surprising, as they were usually hidden in studies of neuromuscular synaptic function, including our own report of spindle labelling with the styryl pyridinium dyes. This, then, was a period of great excitement for us, as trawling the literature uncovered many disparate pieces for an emerging jigsaw puzzle that began to fit together.

## SLV recycling is the basis of FM1-43 labelling

**Fig 3:**
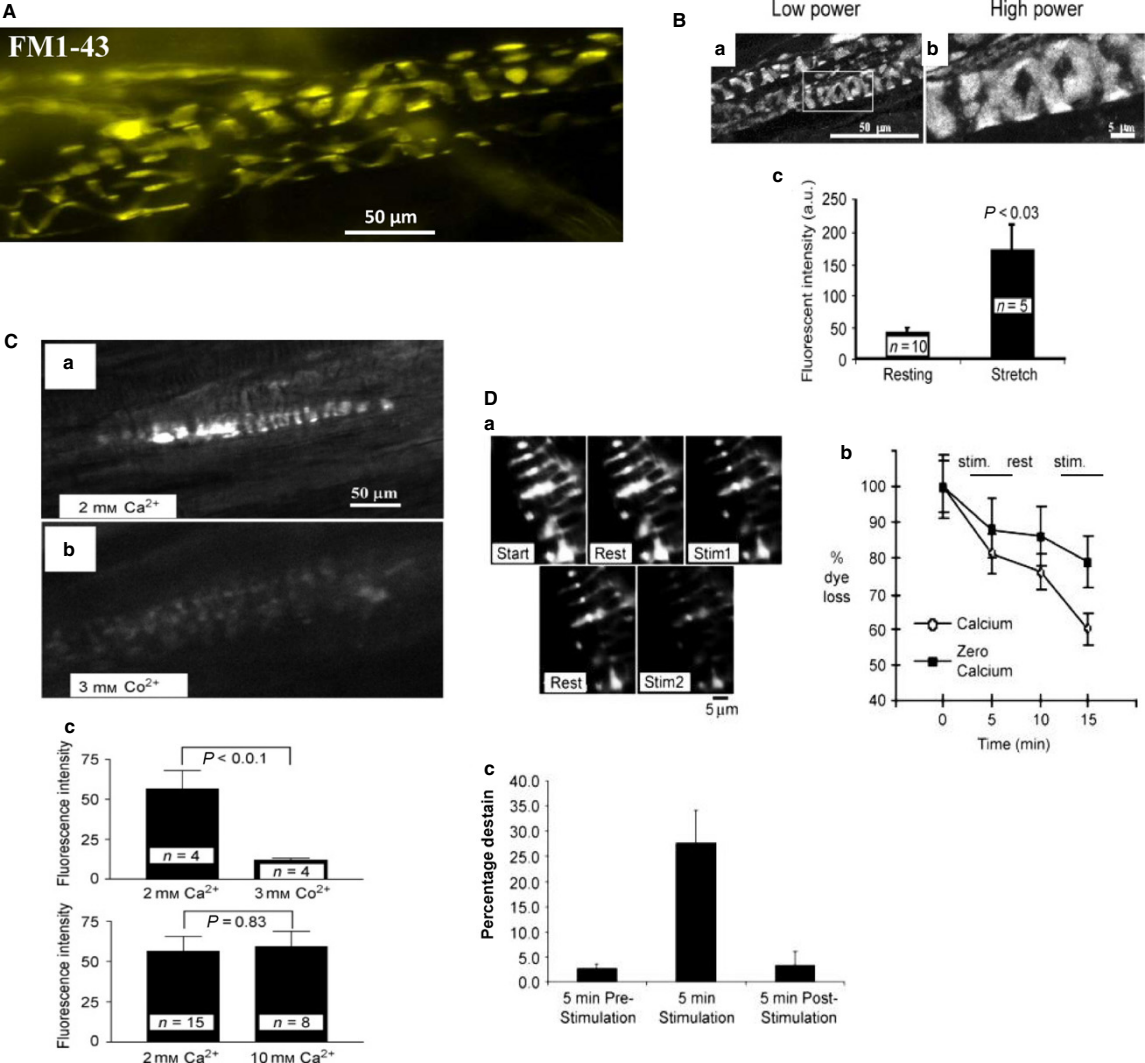
Characteristics of FM1-43 labelling of muscle spindle annulospiral primary sensory endings. (A) FM1-43 labelling of a primary afferent terminal in a live rat lumbrical muscle, *ex vivo*. (B) Maximum intensity projections of a confocal series of optical slices in a rat lumbrical spindle at low magnification (a) and an expansion of the rectangular area is also shown (b). (c) Labelling occurs spontaneously (resting length), but is increased fourfold by stretching (stretch) during incubation (2 h, 10 μm). The muscle was returned to resting length briefly two–three times each 30 min, before being re-pinned at the maximum length. (C) FM1-43 internalisation (a) is strongly inhibited (b) by inorganic salts that block Ca^2+^ channels, in this case Co^2^^+^ . This is quantified below (c, top). Note that 10 mm Ca, which blocks some stretch-sensitive channels, has no effect on FM1-43 internalisation, suggesting it is not entering through the mechanosensory channels, but rather by SLV endocytosis. (D) This conclusion of vesicle-mediated labelling is further reinforced by the release of dye from labelled terminals. This is in sharp contrast to labelling by the dye permeating through the pore of the open mechanosensory channels, which is irreversible. (a) A labelled terminal (Start) shows little dye loss during 5 min rest (Rest). However, 5 min of vibration (200 Hz, 50 μm) applied to the pole of the spindle with a blunt vibrating probe elicits a marked reduction of intensity, i.e. dye loss (Stim1). The rate of destain returns to basal levels on returning to rest, but resumes on a second vibration (Stim2). This indicates FM1-43 is being lost by SLV exocytosis, and at a rate proportional to the mechanical activity. (b,c) The vibration-evoked destaining was quantified and was markedly reduced in 0 mm Ca^2^^+^. Thus, destaining (i.e. SLV exocytosis) is Ca^2^^+^ -sensitive, which is another parallel with synaptic vesicle turnover. (B–D) From Bewick et al. (2005) with permission.

So, this is the point where our own experiments into SLV function began. Why not start by asking if SLVs do indeed undergo recycling, using the styryl pyridinium dyes I’d help develop 10 years earlier? Our 1992 report used the orange/red dye RH414, so now we first asked if the more widely used yellow/green FM1-43 was also internalised – which it was (Fig.3A). The next studies asked how ‘synaptic-like’ was the labelling functionally, and again reinforced the synaptic similarities – but also revealed some significant differences.

**Fig 4:**
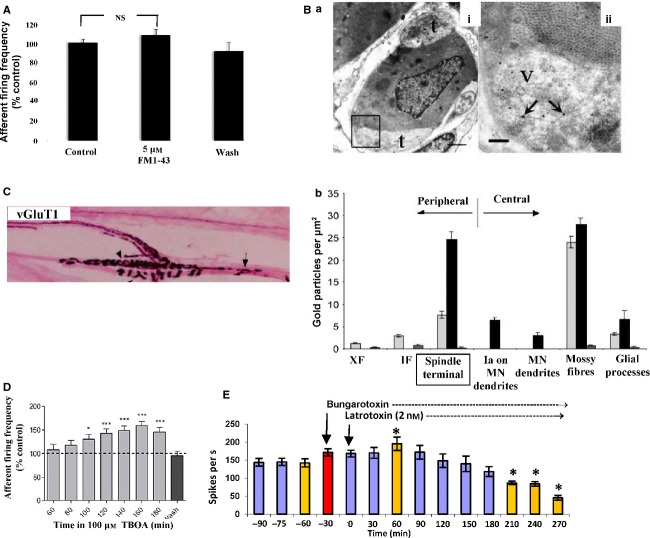
FM1-43 does not block stretch-evoked spindle firing and evidence that endogenous glutamate secretion from SLVs regulates spindle stretch sensitivity. (A) 2 h in 5 μm FM1-43 does not inhibit stretch-evoked spindle firing in rat 4th lumbrical muscles, indicating the dye does not block the mechanosensory channels in muscle spindles and, therefore, terminal labelling is unlikely to be due to dye permeation through the channels expressed in these fully differentiated mature terminals. (B) Immunogold labelling for glutamate in muscle spindle primary afferents. (a) Transverse ultrathin section through an intrafusal fibre (i) with two paler terminal profiles (t) on its surface, labelled with immunogold for glutamate. (ii) Higher magnification of the rectangular area in (i) showing the high density of gold particles in vesicle (v) containing areas of the terminal compared with surrounding structures, including the intrafusal muscle fibre. For this technique fixation is milder, to preserve antigenicity, so vesicle preservation is not as clear in these sections. (b) Quantification of gold particle density of two different spindle Ia primary terminals compared with other tissues in the same rat (dark and light grey bars, respectively) and no primary controls (small mid-tone grey bars). In both cases, glutamate-like immunoactivity (gold particle density) was at least twice that in non-glutamatergic tissues, such as glial cell processes, intra- and extrafusal muscle fibres and motor neurone dendrites. It was also at least as much as in putative central terminals of I afferents on motor neurone dendrites. In one case (dark bars) it was equivalent to that in cerebellar mossy fibre terminals of the cerebellum (both are glutamatergic synapses). No primary controls show negligible labelling. IF, intrafusal fibres; XF, extrafusal fibres. From Bewick et al. (2005), with permission. (C) Spindle primary endings label heavily for the vesicular glutamate transporter vGluT1, indicating the endogenous glutamate is loaded into SLVs (from Wu et al. 2004, with permission). (D) Inhibition of glutamate re-uptake with TBOA greatly increases stretch-evoked firing from rat lumbrical muscle spindles over a 2–3-h period, in a reversible manner. This indicates the extracellular accumulation of endogenously secreted glutamate makes the ending more sensitive to stretch. *P < 0.05, ***P < 0.001 vs pre-drug control firing. (E) Latrotoxin application, which causes uncontrolled exocytosis in spindles, substantially increases stretch-evoked spindle firing in rat 4th lumbricals by 1 h of application, presumably as glutamate exocytosis is greatly increased. Over the next few hours, firing to a standard stretch slowly declines, becoming inhibited from 210 min (3.5 h) of toxin incubation. This presumably reflects SLV, and hence glutamate, depletion. Bungarotoxin was added to block interference by the activation of the intrafusal fibres by fusimotor neurones. Red bar = bungarotoxin application. Yellow bars = statistically significant in comparison to *t* − 60 min (pre-drug control) at (*) *P* < 0.01. Thus, *t* + 60 min (latrotoxin peak excitation), *t* + 210–270 min (latrotoxin inhibition).

Indeed, the first observation showed a major difference – that dye uptake occurred spontaneously (Fig.3B). However, like synapses, uptake was proportional to activity. In spindles, however, this was evoked by mechanical activity, whereby repeated stretching increased labelling fourfold. Labelling, like stretch-evoked firing, was Ca^2^^+^ -dependent as it was blocked by replacing Ca^2+^ with Mg^2+^ or Co^2+^ (Fig.3C). Dye was also lost again with vibrational activity, and this was also Ca^2^^+^ -dependent (Fig.3D). Whether the increased dye fluxes are driven by stretch-evoked AP firing, or by mechanical activity alone, is not clear. Nevertheless, these observations show the dye intensity changes reflect endo- and exocytosis of SLVs. This point is important as it is in sharp contrast to labelling in mechanically stimulated cochlear hair cells and in neurites of dorsal root ganglia (DRG) neurones in culture (Gale et al. 2001; Drew & Wood, 2007). In these preparations, dye enters irreversibly by permeation of mechanosensory channels, a process blocked by elevated external Ca^2+^ (10 mm), which blocks the mechanosensory channels in these preparations (Ricci & Fettiplace, 1998; McCarter & Levine, 2006). Upon entry, the dye subsequently blocks the channels (Gale et al. 2001; Drew & Wood, 2007). In contrast, our observations in mature spindle terminals differed in all three significant respects. Spindle dye internalisation was not inhibited by elevated Ca^2+^, was reversible and dye did not block stretch-evoked firing at 5 μm (Fig.4A). Even after 3 h at 10 μm, routinely used by us to label the endings (Fig.1A), firing was only reduced by 30% – and this too was reversible. In DRG neurites, this concentration very rapidly produces an 80% block. Thus, the labelling of mature, fully differentiated afferent endings seems at least predominantly due to SLV endocytosis and has a different basis to that in neonatally derived tissues.

## SLVs secrete glutamate, maintaining stretch-sensitivity

**Fig 5:**
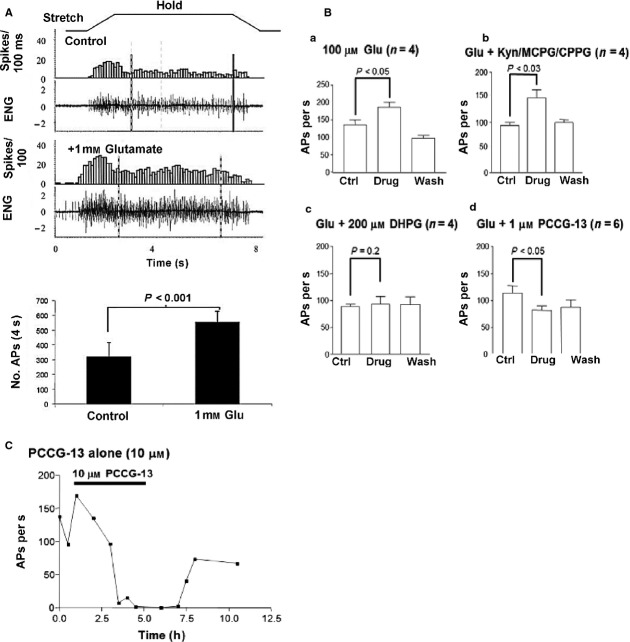
Spindle stretch sensitivity is regulated by an atypical glutamate receptor with the pharmacology of the hippocampal PLD-mGluR. (A) (Top to bottom). A trapezoid profile of the stretch applied to the rat 4th lumbrical muscles. Muscles are stretched by 1 mm, which represents ∼10% increase in length, for 5 s before returning to the original length. The ‘spike rate’ is shown in 100-ms windows, revealing a rapid increase in firing for this particular muscle during the stretch to a new length (dynamic response). Firing then settles to a slightly lower plateau rate at the new length, until released to the original length, when firing stops. The ‘afferent discharge’ is shown in the raw electroneurogram, which is recorded from the whole muscle nerve, and thus represents the firing from all the 8–12 spindles found in this lumbrical muscle. Below this, the same muscle response is shown following 1 h incubation in 1 mm glutamate. This approximately doubles the firing rate for the same stretch. The very bottom histogram shows quantification for *n* = 6 preparations. This increase in stretch-sensitivity is entirely reversible (not shown). (B) The reversible increase in firing rate with 100 μm glututamate (a). (b) This response cannot be blocked by antagonists of all the 11 cloned glutamate receptors (kynurenate: all three iGluRs; MCPG/CPPG: all eight mGluRs), even when applied together. This indicates glutamate is not acting through any of the cloned receptors. (c) Glutamate excitability is totally blocked by *RS* 3,5-DHPG, an agonist at group I mGluRs but an antagonist of the PLD-mGluR first reported in the hippocampus. (d) Glutamate excitability is blocked somewhat more potently by PCCG-13, a selective antagonist specifically developed for this receptor. These experiments indicate exogenous glutamate is activating the PLD-mGluR to regulate spindle stretch-sensitivity. (C) Quantification of stretch-evoked firing in a single rat 4th lumbrical muscle during prolonged PCCG-13 application. When applied alone, without exogenous glutamate, high concentrations (10 μm) of the selective PLD-mGluR antagonist PCCG-13 can totally abolish stretch-evoked responses, over a period of 4–6 h. This effect is entirely reversible. This illustrates that blocking the activation of the PLD-mGluR by endogenous glutamate secretion from SLVs means the ending cannot sustain a sensitivity to stretch, i.e. endogenous glutamate-mediated activation of the receptor is necessary to maintain its stretch sensitivity. From Bewick et al. (2005), with permission.

We next reasoned that if SLVs undergo exocytosis, they should presumably store and release a neuroactive chemical of sorts, and sought to identify it. Using Dale’s Principle (Dale, 1935) that a neurone secretes the same neuroactive substance at all of its terminals, and knowing that the central terminals of primary afferents are glutamatergic (Engberg et al. 1993; Walmsley & Bolton, 1994) we, with excellent technical assistance from Christine Richardson, used immunogold EM to show there was indeed glutamate-like immunoactivity in annulospiral endings, and at levels equivalent to central synaptic Ia endings, or other glutamatergic central synapses (Banks et al. 2002). Again, this finding was supported 2 years later by a report that primary afferent terminals expressed a transporter specific for loading glutamate into vesicles (i.e. vGluT1, although not vGluT2 or 3; Wu et al. 2004). The next obvious question was, if SLVs secreted glutamate, what role secretion might play. We found exogenous glutamate enhanced stretch-evoked firing (Fig.5) in a dose-dependent manner (up to 1 mm), supplementing the previous experiments by Mizote & Takano (1985) showing blocking SLV exocytosis with tetanus toxin profoundly inhibited firing. More recent experiments (by Anna Simon, postdoctoral research fellow) have produced more evidence that SLVs secrete endogenous glutamate, as TBOA (dl-threo-beta-benzyloxyaspartate), the competitive, non-transportable glutamate re-uptake inhibitor strongly increases spindle excitability (Fig.4D), while latrotoxin, which potentiates SLV exocytosis to the point of total depletion (Queiroz & Duchen, 1982), initially enhances, then inhibits stretch-evoked firing (Fig.4E).

There are three things that all these manipulations have in common that suggest they involve a common target. All enhancements are to a maximum of 75–100%, they take ∼1 h to become significant, and take several hours to reach a maximum. Thus, all these modulations are long term. While some of this delay is undoubtedly due to the physical and physiological barriers to drug access (penetration past first the surrounding extrafusal muscle fibres, then the selectively permeable spindle capsule), the protracted time-course even after drug penetration suggests delayed penetration is not the only factor. Rather, it implies the ultimate target is a metabotropic glutamate receptor (mGluR) and these effects are second-messenger mediated.

However, identifying this glutamate receptor has proven quite a challenge, as this glutamate-stimulated excitation was not blocked by the classical antagonists of either ionotropic glutamate receptors (iGluRs) or mGluRs, whether applied singly or simultaneously as one large cocktail at supramaximal concentrations (Bewick et al. 2005). This engendered another trawl through the literature. Here we unearthed a little known mGluR linked to phospholipase D (PLD) activation, first reported in the hippocampus (Boss et al. 1994; Pellegrini-Giampietro et al. 1996). This receptor was actually inhibited by the classical agonist for group I mGluRs *R*,*S* 3,5-DHPG, and rather more effectively by a specific antagonist PCCG-13, the only one of 16 isoforms tested (PCCG-1 to 16) that produced such profound inhibition in this hippocampal receptor (Albani-Torregrossa et al. 1999). As in the hippocampus, we found glutamate-stimulated spindle excitation was inhibited by PCCG-13 or *RS* 3,5-DHPG or by PLD inhibition (using FIPI; Monovich et al. 2007) (Fig.5B). Strikingly, PCCG-13 applied in the absence of glutamate could abolish stretch-evoked spindle firing entirely when applied at high concentrations (10 μm) for long periods (4 +  h), an effect that was entirely reversible (Fig.5C). This is important as it implies that the constitutive SLV-mediated glutamate secretion revealed by spontaneous FM1-43 uptake, is to ensure tonic PLD-receptor activation and this in turn is necessary to maintain the spindle’s ability to respond to stretch.

## Implications for the role of SLV-mediated glutamate secretion

If our interpretations of these observations concerning SLVs are correct, they lead to a number of quite interesting conclusions. First, SLVs undergo tonic exocytosis. Terminals must therefore continuously release glutamate. Second, as dye uptake is increased by stretch, the rate of SLV recycling (and presumably glutamate secretion) is accelerated by activity. Third, PLD-mGluR antagonists and transporter inhibitors applied alone are only regulating responses to this tonic endogenous glutamate release. The strong inhibition by PLD-mGluR antagonists implies tonic receptor activation by endogenous secretion is required to maintain spindle stretch sensitivity. Conversely, because the TBOA-induced sensitivity increase is as great as for any exogenous ligand, it implies endogenously released glutamate can stimulate as effectively as any exogenously applied ligand. Fourth, this system has evolved to work over the extremely long term. It takes at least 1 h to significantly change stretch-evoked firing, and often several hours to reach maximal effect. Therefore, it seems very unlikely the stretch-activated secretion of glutamate is the first step in the gating of mechanosensory channels that produce the RP in mechanotransduction. Rather, the SLV/PLD-mGluR system seems more important for long-term regulation of terminal stretch sensitivity. I tend to think of this control system using a radio analogy; the transduction channels of the classical system are the ‘on/off’ or ‘power on’ button, while the SLV/glutamate/PLD-mGluR system acts as the ‘volume’ control.

However, these experiments leave two fundamental questions unanswered. Where is the PLD-mGluR located? And, how does its activation lead to increased ending stretch sensitivity? These are the subjects of ongoing research in the laboratory. Sonia Watson’s article in this volume describes the progress we are making towards developing tools to isolate and label the receptor protein. This will hopefully help us to make substantial progress towards answering at least the first of these questions. Possible mechanisms by which the second might be achieved include increasing the channel open probability or their abundance in the membrane. The latter would be consistent with the regulation of ENaC (epithelial sodium channel) activity in the kidney cortical collecting duct, where they are stored in sub-plasmalemmal vesicles and recycled into/out of the membrane, with a half-life of 20–120 min (Butterworth, 2010). I raise ENaCs at this juncture as they are candidate mechanosensory channels (see below).

## SLVs, glutamate secretion and PLD-mGluRs: a general principle of mechanosensory endings

This seems an appropriate point to look at the generality or otherwise of this glutamatergic regulatory system. One of the earliest thoughts Bob and I had on reviewing the literature was that all primary mechanosensory endings examined at the on ultrastructural level had been reported to have SLVs, i.e. clear, 50-nm-diameter vesicles (Bewick et al. 2005). This, in turn, implied the glutamatergic signalling should be a ubiquitous feature of such endings. We should, therefore, find evidence of glutamate signalling and SLV turnover in other primary mechanosensory endings. And so, we chose two other, quite diverse systems to test this hypothesis – aortic baroreceptors that monitor blood pressure, and lanceolate endings from skin hair follicles (Paton et al. 2010; Banks et al. 2013). Lanceolate endings can be very rapidly adapting, in contrast to spindle annulospiral endings, i.e. firing only during movement and not sustaining firing for a maintained deflection of the hair. Thus, they might prove an informative contrast to the very slowly adapting spindle endings. Furthermore, we had been told by our mutual long-term friend Prof Clarke Slater (see below), that these endings took up FM dyes much more rapidly than spindle endings, and so might be a useful preparation to examine the pharmacology of SLV recycling. We chose to examine baroreceptor terminals to see if aspects of this new system, if present, might have translational potential. We felt that in baroreceptors the glutamate signalling system may be a new target for controlling blood pressure in hypertensives.

**Fig 6:**
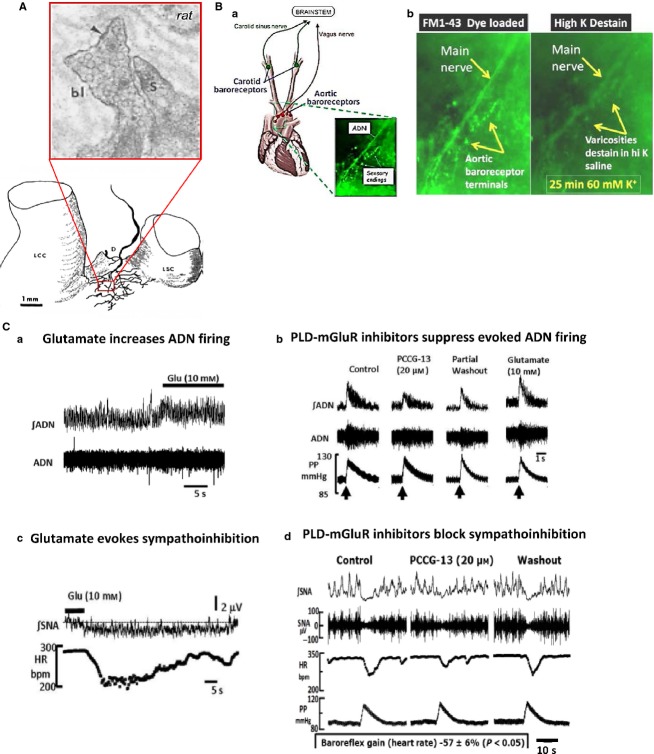
Baroreceptor terminals have SLV, internalise and release FM1-43, and exhibit glutamate sensitivity for stretch-evoked firing. (A) Baroreceptor terminals on the aortic arch of the rat (lower image) have a high density of SLVs, when viewed at the EM level. D, aortic depressor nerve; LCC, left common carotid artery; LSC, left subclavian artery. (B) Aortic baroreceptor terminals take up and release FM1-43. Adapted from Krauhs (1979), with permission. (a) Schematic of the anatomical position of the aortic baroreceptors in humans. Inset, FM1-43 labelling of baroreceptor terminals in mouse. (b) Higher magnification of FM1-43-labelled baroreceptor terminals shown in the inset in (a). Terminal depolarisation with 60 mm K^+^ stimulates FM1-43 release, indicating dye internalisation and release is due to SLV recycling. (C) Responses of various working heart brainstem preparation outputs to topical glutamate application to the baroreceptors. (a) Glutamate application to the aortic baroreceptors increases firing rate of the aortic depressor nerve (ADN). *∫ADN*, integrated ADN activity. (b) Increasing (arrow) perfusion pump pulse pressure (PP) in the aorta evokes a marked increase in ADN firing. Topical PCCG-13, the selective PLD-mGluR antagonist, onto the baroreceptor greatly reduces the evoked response, an effect that can be washed out. Subsequent exogenous glutamate application increases pressure-evoked ADN firing again. (c) Topical glutamate application to the baroreceptors produces a dramatic decrease in heart rate, and sympathetic nerve firing. Thus, enhancing baroreceptor sensitivity causes reflex inhibition likely to induce a reduction in peripheral blood pressure. bpm, beats per minute; HR, heat rate; ∫SNA, integrated sympathetic nerve activity. (d) Just as topical glutamate application to the aortic baroreceptors increases sympathoinhibition, PCCG-13 reduces it. These experiments show the PLD-mGluR on baroreceptor terminals can be a suitable target for regulating sympathoinhibition, which controls peripheral blood pressure.

Baroreceptor terminals are packed with SLVs (Fig.6A; Krauhs, 1979), so in collaboration with Julian Paton in Bristol, we sought evidence for a role for the PLD-mGluR in baroreceptor function. We found that aortic baroreceptors in the isolated aortic arch take up and release FM1-43, i.e. the SLVs recycled locally, undergoing endocytosis (dye uptake). The subsequent exocytosis (dye release; Fig.6B) again ruled out this labelling was by direct permeation of the mechanically gated channel by the dye. Using Julian Paton’s working heart brainstem preparation (Paton, 1996), we studied the effect of topical glutamate application on baroreceptor outputs and feedback pathways (Paton et al. 2010). Glutamate application to the baroreceptors increased aortic depressor nerve discharge, while PCCG-13 inhibited it (Fig.6D). Moreover, this evoked reflex inhibition of the heart rate and increased sympathetic nerve activity (SNA; Fig.6Dc). Sonia Watson, a PhD student in my laboratory, has now shown in this same model that other PLD-mGluR ligands applied to baroreceptor endings also modulate SNA in a predictable manner – agonists inhibit SNA, while antagonists enhance it.

**Fig 7:**
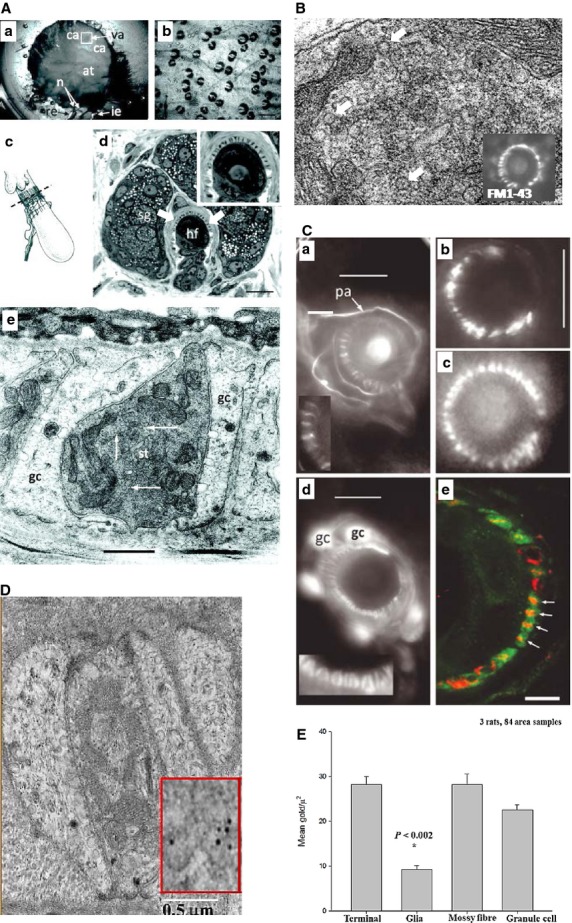
Hair follicle lanceolate mechanosensory endings of the anterior skin of the mouse pinna have SLVs, secretion-associated proteins and glutamate. (A) (a) The mouse pinna preparation pinned, anterior skin face down, in a Sylgard-lined dish filled with Liley’s solution, and set up for electrophysiological recording. The whole posterior skin has been removed, as well as a large area of elastic cartilage and adipose tissue (at) from the cleared area (ca). By folding the cleared area back, access was gained to the hair shafts, allowing two or three within the vibrated area (va) to be mechanically displaced by a fire-polished glass capillary (not shown). The nerves (n) are branches of the mandibular division of the trigeminal and are set up for differential recording of the neurogram using recording (re) and indifferent (i.e.) suction electrodes. (b) Brightfield image of mouse pinna skin viewed from the dermal side, showing several hair follicles. The bases of the hair shafts are clearly seen; each shaft is partly surrounded by a sebaceous gland that appears dark. Scale bar: 100 μm. (c) Diagram of the structure and location of the innervation of a hair follicle. The lanceolate ending consists of the group of terminals forming the palisade-like structure immediately below the lobular sebaceous gland (from Bannister, 1976). The dashed line indicates the typical plane of section for subsequent images for fluorescence and light microscopy. (d) Semi-thin (1 μm) cross-section through a hair follicle (hf) at the level of the sebaceous gland (sg) and lanceolate ending, as indicated in (c). The lanceolate ending surrounds the follicle (arrows), and terminals appear as dark structures alternating between lighter accessory cells, shown in greater detail in the inset at top right. Mouse pinna, Toluidine Blue; scale bar (main image) indicates 20 μm. (e) EM of an ultrathin cross-section through a lanceolate ending, showing a single, darkly stained, sensory terminal (st) almost completely enclosed by pale-staining glial cell (gc) processes. Note the numerous 50-nm-diameter vesicle profiles in the terminal axoplasm (white arrows). Mouse pinna; scale bar indicates 0.5 μm. (B) SLVs shown in higher magnification (white arrows), and labelling with FM1-43 produces a characteristic circle of lanceolate endings around a central hair shaft. (C) Immunohistochemical and genetic identification of the sensory terminals and glial cells of lanceolate nerve endings. (a) Anti-neurofilament protein (NFP)-like immunoreactivity is localized in structures identified as preterminal axons (pa) and sensory terminals in a mouse pinna follicle. Several terminals are shown enlarged in the inset. Epifluorescence; scale bar indicates 10 μm. (b) Structures identified as sensory terminals also react strongly with anti-synapsin I antibody. Mouse pinna, epifluorescence; scale bar indicates 20 μm. (c) SynaptopHluorin fluorescence shows the expression of the v-SNARE synaptobrevin in the lanceolate terminals in a very similar pattern to NFP and synaptophysin. Mouse pinna, epifluorescence; scale bar as in (B). (d) Anti-S-100 antibody, in contrast, labels paired structures identified as glial cells (gc) and their processes in a mouse pinna follicle. Pairing of the processes is particularly apparent in the enlarged inset and is distinct from the unpaired processes seen in (a). Epifluorescence; scale bar indicates 10 μm. (e) A follicle from rat pinna double-labelled with antibodies against synaptophysin (red) and S-100 (green). Where the ending is precisely orthogonal within the section (white arrows), individual red profiles can be seen clearly to be almost entirely enclosed by paired green profiles, identified as sensory terminals and glial cell processes, respectively. Laser-scanning confocal microscopy; scale bar indicates 5 μm. (D)Lanceolate sensory terminals are enriched in glutamate. An EM of a thin section of a sensory lanceolate terminal (st) is shown with enclosing glial cells (gc) immunogold labelled to show glutamate-like immunoreactivity. A portion is enlarged in the inset, showing gold particles more clearly. (E) A histogram (means ± SEM) summarising the quantitative assessment of glutamate-like immunoreactivity. From Banks et al. (2013), with permission.

As noted briefly above, lanceolate terminals are mechanosensory endings detecting hair movements in hair follicles of the skin (Fig.7A,B). Professor Clarke Slater (Newcastle University) developed a mouse ear skin preparation, and showed the terminals contain SLVs and they readily internalised FM1-43 (Kain & Slater, 2003). Our four laboratories [Clarke Slater, Bob Banks and myself, together with Peter Cahusac (then University of Stirling, now Alfaisal University, Saudi Arabia)] subsequently found the terminals immunolabelled for the synaptic vesicle proteins synapsin I and synaptophysin, expressed synaptobrevin (synaptopHluorin) and synaptic levels of glutamate, the SLVs underwent local recycling (internalising/releasing FM1-43) and latrotoxin accelerated destaining (Banks et al. 2013). FM1-43 had no effect on afferent firing either, as further evidence that dye uptake was not due to internalisation through mechanosensory channels. Thus, lanceolate preparations proved extremely convenient for studying SLV recycling: there are large numbers of follicles per preparation (Fig.7A) and dye uptake is rapid (∼30 min vs. 120 min for spindles). They were, therefore, an ideal preparation to explore how SLV recycling is regulated.

## Regulation of SLV recycling

**Fig 8:**
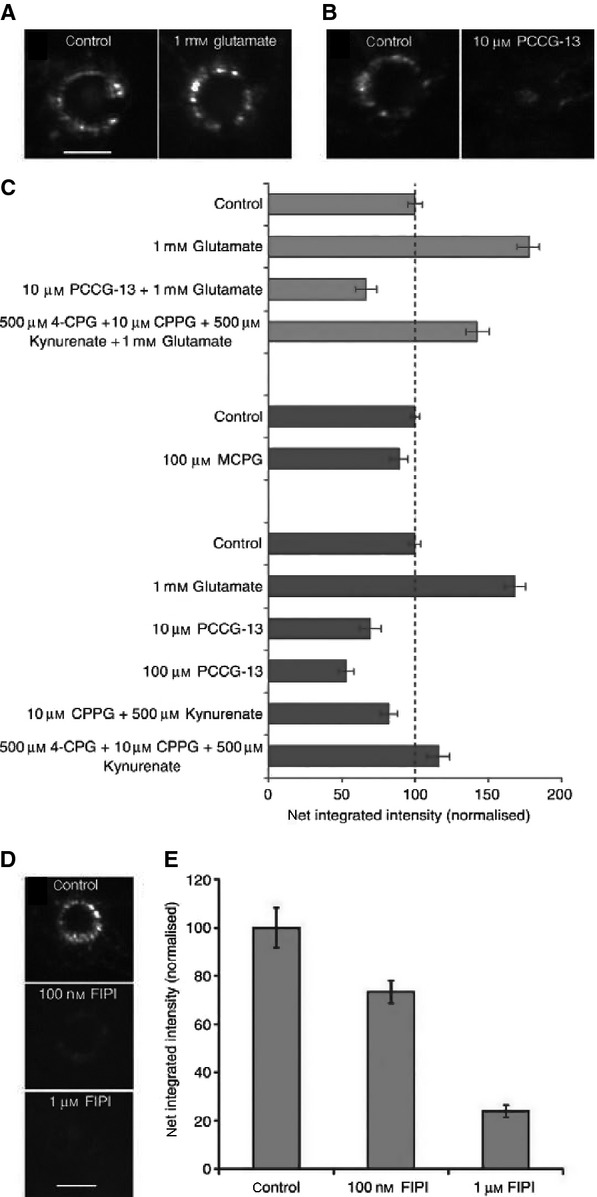
Glutamate regulates SLV recycling by activating the PLD-mGluR. (A) Like spindle afferent terminals, lanceolate endings spontaneously label with FM1-43. Exogenous glutamate increases, while (B) PCCG-13 decreases, dye internalisation. (C) Top to bottom. Histogram summarising the effects of various GluR ligands on FM1-43 internalisation. Light grey bars – the glutamate-mediated increase is blocked by PCCG-13 but not the cocktail of classical iGluR and mGluR antagonists (4-CPG, CPPG, kynurenate). Dark grey bars – MCPG (group I and group II antagonist) does not significantly inhibit FM1-43 internalisation, while glutamate increases it, and PCCG-13 produces a dose-dependent decrease. The other antagonist cocktails produce little if any effect on labelling. (D) FIPI, the PLD inhibitor, also causes a dose-dependent decrease in FM1-43 uptake/SLV endocytosis. These effects are quantified in (E). These data indicate that, as for spindle firing, the PLD-mGluR regulates SLV endocytic uptake of FM1-43. From Banks et al. (2013), with permission.

Like spindle annulospiral endings, we found Co^2+^ reduced dye uptake by ∼90%, while 10 mm Ca^2+^, which blocks FM1-43 internalisation through mechanosensory channels (Nishikawa & Sasaki, 1996; Gale et al. 2001), had very little effect on labelling (Banks et al. 2013). Unlike synaptic terminal vesicles, SLV turnover showed no dependence on the two major voltage-gated Ca^2+^ channels responsible for synaptic transmission, the N- or P/Q-type channels. The L-type Ca^2+^ channel blockers nifedipine and taicatoxin did reduce labelling. However, this was only by ∼50%, indicating they are important but that there are still other Ca^2+^ sources involved in supporting SLV recycling. Conversely, glutamate increased, while PCCG-13 decreased, labelling markedly. PCCG-13 also completely blocked the glutamate-stimulated uptake (Fig.8A,B). Again, like spindle firing, classic iGluR (kynurenate) and mGluR (MCPG, 4-CPG, CPPG) antagonists had little effect on labelling, and did not block glutamate-mediated stimulation, even when all were applied together (Fig.8C). This cocktail should block all of the glutamate receptors that have been cloned. These data suggest, therefore, that like spindle afferent discharge, PLD-mGluRs regulate SLV recycling, a deduction further supported when we found PLD inhibition (with FIPI) reduced labelling by 80% (Fig.8D,E).

## Ca^2^^+^ -activated potassium channels and candidate mechanosensory channels

**Fig 9:**
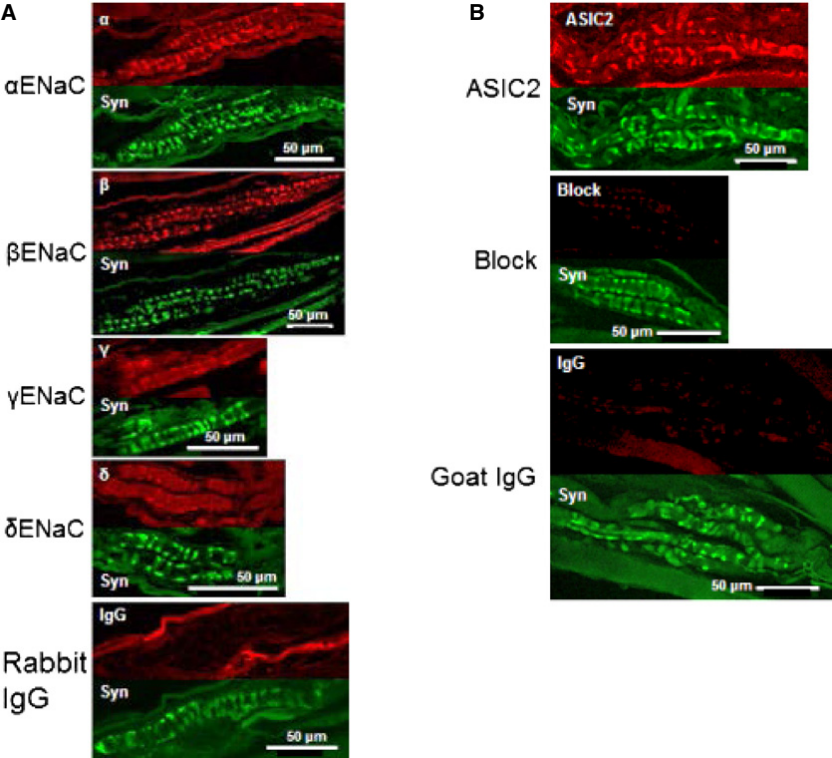
Anti-ENaC and anti-ASIC 2 subunit immunoreactivity localises to sensory terminals of rat muscle spindles. (A) Double-immunofluorescent labelling of the sensory regions of rat muscle spindles, comparing anti-ENaC subunit with anti-synaptophysin reactivities. Upper panels (red): anti-ENaC α, β, γ or δ immunoreactivity; lower panels (green): anti-synaptophysin immunoreactivity of the corresponding spindles. Control: anti-ENaC antibody replaced with non-immune rabbit IgG. Immunoreactivity was clearly visible with antibodies to the α, β and γ subunits, but there was little reaction with the anti-ENaC δ antibody; this is in contrast to the control where no specific reactivity was discernible. (B) Double-immunofluoresent labelling of the sensory regions of rat muscle spindles, comparing anti-ASIC2 with anti-synaptophysin reactivities. Upper panels (red): anti-ASIC2 immunoreactivity; lower panels (green): anti-synaptophysin immunoreactivity of the corresponding spindles. Anti-ASIC2 immunoreactivity was evident on sensory terminals in contrast to controls (anti-ASIC2 antibody blocked with peptide, or replaced with non-immune goat IgG) where specific immunoreactivity of the sensory terminals was not visible. From Simon et al. (2010), with permission.

**Fig 10:**
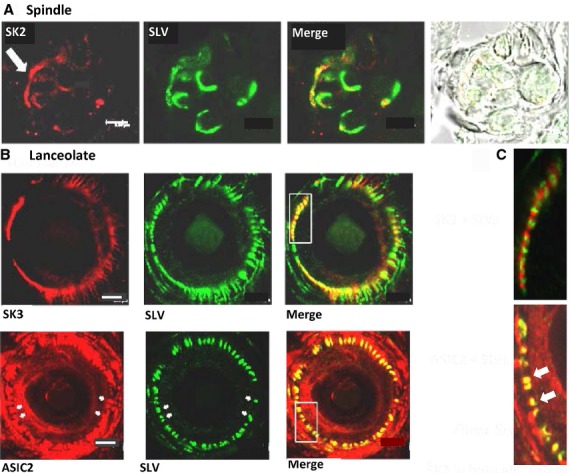
Spindles and lanceolate endings express SK2 Ca^2^^+^ -activated K^+^ channels and ASIC2. (A) SK2-like immunoactivity (red) is present in preterminal axons and the terminals, identified by SLV-associated synaptophysin antibodies (green). The merge shows the colocalisation of the two antibody distributions, while the widefield brightfield image shows the disposition of the intrafusal fibres. Lanceolate terminals also express SK2 (not shown). (B) (Upper row) Just like spindle terminals (not shown), SK3-like immunoactivity (red) is not found in lanceolate terminals (green). In hair follicles, however, SK3 is found in the glial cells enclosing the lanceolate endings [merge and in (C) the higher magnification inset]. (Lower row) Conversely, ASIC2 immunolabelling (red) co-localises with SLV label (green) – see yellow in merge and indicated by arrows in inset in (C). Scale bar: 20 μm (A); 5 μm (B). From Shenton et al. (2014), with permission.

The most recent set of experiments Bob and I have undertaken together, which are still very much ongoing and yet to reach firm conclusions, concern understanding the channels underlying the RP. In a seminal paper, Hunt et al. (1978) reported the major ion contribution to the RP’s initial dynamic current on stretching a muscle spindle is Na^+^, at ∼80%, with Ca^2+^ contributing a further ∼20%. Various K^+^ channels seemed to be responsible for the repolarisation when movement stopped at the new length and the hyperpolarisation on returning back to the original length. The articles by Bob Banks and Zhuoyi Song in this volume discuss this complex potential waveform in more detail. Here, I will briefly summarise our findings regarding candidate channels involved. Given the preponderance of Na^+^ in the ionic basis of the RP, we have been examining Na^+^-selective candidate mechanosensory channels, particularly members of the degenerin (DEG)/ENaC family. Homologues of the DEG/ENaCs have been identified as the stretch-sensitive channels in primary mechanosensory nerve terminals in the nematode worm *Caenorhabditis elegans* (reviewed by Arnadóttir & Chalfie, 2010). We tested for both ENaC itself and also the closely related acid-sensing ion channels (ASICs). This work was undertaken with two very talented postdoctoral workers, Fiona Shenton (in Durham) and Anna Simon (in Aberdeen). In 2010, using spindles, we reported Western blotting, immunolabelling of whole-mount preparations and amiloride-sensitive inhibition of stretch-evoked firing consistent with ENaC and ASIC2a channels underlying these currents (Fig.9A,B). In lanceolate endings we have not yet looked for ENaC, but we again find ASIC2 (Fig.10; Shenton et al. 2014), while other studies report ASICs and ENaC subunits in baroreceptors (Drummond et al. 1998; Lu et al. 2009). To date, the functional importance of these channels in spindles awaits validation in genetically modified animals. ENaC knockout is lethal, due to its great physiological importance in kidney function and has not been studied in mechanosensory function, while ASIC knockout studies, even triple knockouts (Kang et al. 2012; Gautam & Benson, 2013), reveal at most modest perturbance of mechanosensation in touch afferents. Interestingly, most other mechanosensory channel candidates (e.g. transient receptor potential (TRPs), reviewed in Arnadóttir & Chalfie, 2010; and Piezos, see Ranade, Woo, et al. 2014) are non-selective cation channels, passing Na^+^ and Ca^2+^ equally or with a Ca^2+^ bias. This alone, at least at a simplistic level, seems to exclude them as likely candidates to underlie the spindle RP. Despite this, Piezo2, a non-selective cation channel, is currently the leading candidate as a transducer of many aspects of touch mechanosensation (Ranade et al. 2014), so further work is clearly needed in this area to explain these apparently conflicting findings. However, we have strong evidence for Ca^2^^+^ -activated K^+^ channels in both spindles and lanceolate terminals. Both types of terminal express SK2-type channels, while SK3 seems to be mainly in the glial cells surrounding the lanceolate terminals and is absent from spindle endings (Shenton et al. 2014). This is further evidence of commonality of properties in these types of ending. These channels seem likely to be responsible for at least some of the K^+^ currents Hunt et al. identified in the stretch-activated RP.

## Summary and musings on the nature of the PLD-mGluR

**Fig 11:**
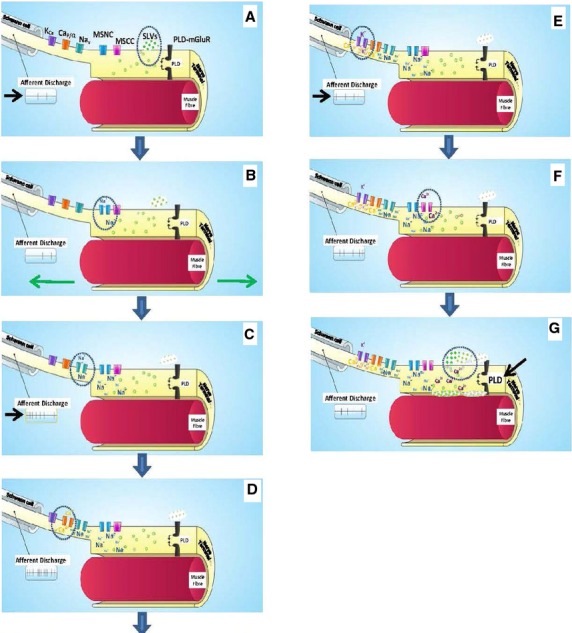
Emerging model of stretch-evoked firing in muscle spindles. Cartoon schematic showing the steps in the processes evoked by stretch in a muscle spindle afferent. The points of interest at each step are circled while changes in afferent discharge rate are indicated by the arrow. (A) Tonic secretion of glutamate from SLVs maintains stretch sensitivity of the ending via low-level activation of the PLD-mGluR, and a low, tonic firing at rest. (B) Stretch opens a mechanosensitive Na^+^ channel, producing the depolarising RP. (C) This, in turn, opens the voltage-gated Na^+^ channels in the AP initiation site, probably in the first heminode (see Cope article in this volume), greatly increasing the afferent discharge rate. (D) The depolarisation also opens voltage-gated Ca^2+^ channels, activating SK2 Ca^2^^+^ -activated K^+^ channels (E). The resulting K^+^ efflux repolarises the membrane, reducing the afferent discharge rate to more modest levels. (F) Meanwhile, in the terminal, stretch also opens a Ca^2+^ channel, which enhances SLV exocytosis (G), increasing the PLD-mGluR activation. How this maintains ending sensitivity is not clear at present, but may be through regulating mechanosensory Na^+^ channel insertion from the vesicle store. These may be the same, or a different, pool to the SLVs. From Bewick & Banks (2015), with permission.

**Fig 12:**
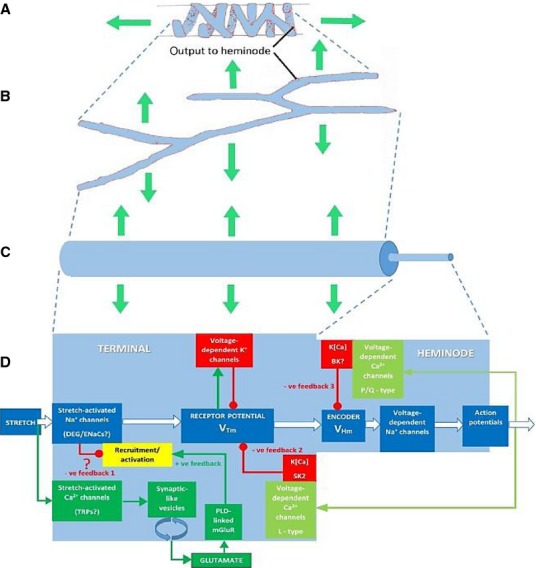
Progressive geometrical abstraction of a single terminal of a spindle primary ending, leading to a flow-chart summarising the events of mechanosensory transduction. Green block arrows in (A–C) indicate the direction and distribution of stretch applied to the terminal when the primary ending is lengthened during muscle stretch or fusimotor stimulation. (A) A single terminal in its annulospiral form, taken from a primary ending reconstructed from serial sections. Several such terminals typically enclose a single intrafusal muscle fibre. The terminal is connected to its associated heminode by a short, unmyelinated preterminal axonal branch at the point shown. (B) The terminal unrolled and turned through 90 °. Note that individual terminals may be repeatedly branched and that the direction of stress during stretch is orthogonal to the long axis of the terminal. (C) A terminal and its associated unmyelinated preterminal branch shown in abstract as cylinders whose diameters indicate the relative diameters of these structures in a spindle Ia primary afferent. The smaller preterminal branch to the right is about 1 μm diameter. The lengths, especially those of the much larger terminal to the left, are highly variable. (D) Flow chart to illustrate the main events of mechanosensory transduction, as described in this review. The principal feedforward pathway from stimulus (stretch) to output (APs) is shown by the white block arrows. We envisage that the overall gain of this pathway is controlled by several feedback pathways: negative feedback 1 is at present hypothetical and is included to account for the reversible silencing of the primary ending by PCCG-13 inhibition of the PLD-linked mGluR; the positive feedback pathway is the well-established SLV/glutamatergic loop; negative feedbacks 2 and 3 involve different kinds of K[Ca], one located in the terminal, the other in the heminode and both perhaps triggered by APs opening voltage-gated Ca channels. Green lines and arrowheads indicate enhancing/excitatory actions; red lines and circles indicate reducing/inhibitory actions. From Bewick & Banks (2015), with permission.

Overall, the studies Bob and I have undertaken together over the last 15 years have uncovered a SLV-based glutamatergic secretory system that seems to be a feature of all mammalian mechanosensory endings studied to date. There is now substantial evidence for a somewhat similar system in Merkel endings, in addition to our own studies in lanceolate endings and baroreceptors. Interestingly, in Merkel endings the secretion is from both the terminal and accessory Merkel cells. The relative importance of these two systems remains to be established (Woo et al. 2012). This SLV-based system therefore seems to be an important adjunct to the classical model of mechanosensation, although why this might be the case is not yet clear. In the classical model, tension gates stretch-sensitive channels in the membrane, triggering ion fluxes that generate the RP. We now propose that, simultaneously, the same mechanical stimulus also triggers increased glutamate secretion from SLVs. Over the course of the next several tens of minutes, the glutamate activates a pathway of events through a highly unusual mGluR linked to PLD activation. At least in spindles this constitutive, activity-modulated autogenic PLD-mGluR stimulation seems to be necessary to maintain the ending’s ability to respond to stimuli. Our studies since have attempted to understand how the SLV/PLD-mGluR system fits into the systems controlling mechanosensory ending responsiveness. Those findings, and associated control mechanisms, have recently been reviewed in detail elsewhere (Bewick & Banks, 2015), and are summarised in Figs11 and 12. We suggest that the SLV/PLD-mGluR system is one of several feedback mechanisms that regulate spindle output, from shaping the complex RP (see articles by Zhuoyi Song et al. and Bob Banks in this volume), to exquisitely fine-tuning the afferent discharge rate for each stimulus in this, the most complicated of sensory organs outside of the central nervous system. It seems likely it is these multiple control networks, including the glutamate autoregulatory system constituent of it, that allow the spindle to respond with exquisite sensitivity to both dynamic and static stimuli over a wide range of lengths and velocities.

As for the future, there are a number of pressing questions we are addressing together. First, what is the receptor – is it a new type of mGluR and, if so, what is its sequence? Second, where is it located in the spindle? And, finally, is there translational clinical potential for targeting the SLV/PLD-mGluR system?

### Is it a new glutamate receptor? Is it metabotropic?

As mentioned above, we are developing tools and techniques to isolate this mysterious glutamate receptor, whose true nature and identity continue to tantalise and fascinate. It is certainly activated by glutamate, so at least at this minimalist level it meets the fundamental criterion of a ‘glutamate receptor’. It has been regarded as a mGluR since its discovery in the 1990s. A major reason for us to regard this designation as still appropriate is the persistent resistance of all glutamate-mediated responses in our mechanosensory endings to the application of 1 mm kynurenate, which should inhibit all known iGluRs. However, the PLD-mGluR pharmacology and what little is known of its intracellular signalling linkage are very different from any receptor, either ionotropic or metabotropic, to have been isolated and/or cloned so far. Moreover, BLAST searches by our laboratory and others have found no unassigned closely related sequences, suggesting there are no iGluR- or mGluR-like receptors waiting to be discovered. So, it could either be a splice variant of a known receptor, or a totally unrelated type of receptor that also happens to be activated by glutamate. Alternatively, we have also considered whether our ‘receptor’ may simply be the mechanosensitive channel(s). After all, most GluR ligands are acids that could affect the activity of ASIC channels, a candidate MS channel family. However, this seems unlikely because all our solutions are buffered and there are no systematic effects of ligands on pH. Moreover, it would not be expected that effects involve such long time-scales, nor necessarily be associated with effects on PLD activity and SLV recycling. An association of receptor activation with one of these may be coincidental, but we think coincidence is unlikely to consistently explain all three (long latency, PLD activity and SLV recycling) simultaneously without the involvement of a metabotropic receptor. However, we continue to keep the option of the receptor being a mechanosensory channel open during our search for the receptor protein, as discussed below.

### Isolating the receptor

Regardless of the true nature of the receptor, there does indeed seem to be a glutamate-activated protein to pursue, and we are now trying to isolate it with the newly developed functionalised ligands. From a lack of efficacy in fluorescence-linked Ca^2+^ oscillation (FLIPR) assays, we have found our new ligands do not activate any of the eight cloned mGluRs, whether expressed in cell lines or neonatal cortical neurones (S. Watson, in preparation). Our most recent evidence is, therefore, that our ligands are highly selective for the PLD-mGluR. Our pharmacological characterisations of this receptor in both lanceolate endings (Banks et al. 2013) and spindles (S. Watson, in preparation) suggest none of the cloned receptors affect firing or SLV turnover, i.e. the PLD-mGluR appears to be the only glutamate receptor involved in regulating terminal responsiveness. The only mGluR reported to be present in spindles is mGluR_5_, whose immunoreactivity is detected in fine nociceptor fibres passing through spindles (Lund et al. 2010). However, this seems unlikely to be responsible for the effects we report. First, the synaptic circuitry required for nociceptor-mediated activation is not present (our spindle and lanceolate experiments use excised tissue detached from the spinal cord); second, the pharmacology of mechanosensory responses is quite distinctive from that of classical mGluR_5_; third, nociceptor fibres are not present in all spindles; and, fourth, this would not explain the similar pharmacology in hair follicle lanceolate endings, which do not contain nociceptor fibres.

Collectively, these observations suggest mechanosensory endings themselves are an excellent source of the PLD-mGluR, as they seem uncontaminated by other glutamate receptors. We have recently identified a muscle with a high spindle density (deep masseter muscle) as an enriched receptor protein source. Now, Karen Thompson (a PhD student in my laboratory) is screening spindle homogenates by mass spectrometry, polymerase chain reaction, microarrays, Western and ligand blotting to look both for all the known mGluRs and also for any other gel bands/proteins that might bind our functionalised ligand (ZCZ180, see Sonia Watson’s article). Affinity columns with immobilised functionalised ligand are being used to pull out ligand-binding partners from these homogenates. We are particularly mindful that such binding partners might include mechanosensory channels or other known proteins (e.g. the nociceptor mGluR_5_), and our strategy should be able to directly address these possibilities by protein sequencing of any recovered proteins and cross-validation between recovery methods and molecular weights. We are hopeful, therefore, that this multi-pronged approach will soon yield interesting progress.

### Where is the receptor?

Another big question is which cell type expresses the ‘PLD-mGluR’, given that spindles contain sensory and motor endings, intrafusal muscle fibres, plus inner and outer capsule cells. Applying Ockham’s razor, we have suggested the nerve terminal seems the most parsimonious solution from the available evidence. This is principally because the receptor pharmacology is similar in the hippocampus, spindle, lanceolate ending and baroreceptor. As the only cellular component common to all these diverse tissues is a secretory, usually afferent, nerve terminal, we feel the most parsimonious explanation is that the receptor is on the terminal. Also, the intrafusal fibres do not contract when glutamate is added (C-L Aryiku, M Durand and G.S. Bewick, unpublished observations). However, this does not exclude the possibility the mGluR is indeed on the fibre in spindles, secreting retrograde messengers to act on the terminals when glutamate is applied. Once the receptor isolation and sequencing is achieved, we will raise antibodies to localise the protein unequivocally. For these receptor isolation studies, knowing the cell type expressing the PLD-mGluR is not critical, as the homogenate is made from the whole spindle organ.

### Translational implications?

Reports are emerging that abnormal muscle spindle activity contributes to the pathophysiology of dystonia in spasticity in a variety of conditions, including spinal cord injury (Fukuhara et al. 2010; Rosales & Dressler, 2010; Phadke et al. 2013). Moreover, that as well as the chemodenervation of the extrafusal muscle fibres, locally applied botulinum toxin also blocks activation of the intrafusal muscle fibres, which makes a significant contribution to the anti-spastic effect. Thus, reducing spindle activity in spasticity is a potentially useful new therapeutic target, and inhibiting this highly unusual PLD-mGluR may be a suitable way to achieve the similar effects without resorting to injections of toxins. Finally, following the findings in the working heart brainstem preparation described above, we, together with Julian Paton, will now work to determine if the baroreceptor SLV/PLD-mGluR system might prove a useful new drug target for treating hypertension. High blood pressure is the world’s leading cause of mortality due to the increased risk of stroke, cardiovascular disease and kidney disease (WHO and www.hearstats.org). Chronic baroreceptor stimulation of the carotid sinus directly via implanted electrical devices causes reflex sympathoinhibition, producing substantial (∼30 mmHg) long-term reduction in blood pressure in animal models and even in patients resistant to currently used drugs (Filippone & Bisognano, 2007; Heusser et al. 2010). Unfortunately, implanting electrical stimulators in humans has the considerable disadvantages of the risk, time and expense of invasive surgery, the potential discomfort of gagging reflexes from electrical stimulation in the neck, and the complexity of long-term maintenance and battery replacement (Young et al. 2009). We therefore propose to test the best ligands to target the SLV/PLD-mGluR system of baroreceptors as a potential alternative to electrical stimulation, and we have reported preliminary work in this direction (Paton et al. 2010).

In conclusion, it has been a delight to work on these projects with Bob over the last 15 years: and they all spring from the very simple and unexpected observation of beautiful fluorescent labelling in muscle spindle annulospiral endings. It might be of interest, especially to the younger scientists, that the first 7 years (including most of the observations establishing the principles of the SLV/PLD-mGluR system) received no external funding. Indeed, we were unable to get funding until we had made these observations. We ran our studies purely on our own curiosity and enthusiasm. So, even in the current era, some very interesting science is still possible without funding – although there is no doubt that external grants certainly make it somewhat easier and quicker! Thankfully, our collaboration is set to continue for a number of years yet, and will be dedicated to pursuing the questions set out above. I am very much looking forward to what these studies will uncover and the undoubtedly very enjoyable discussions they will provoke with Bob as he continues his quest to educate this humble synaptic physiologist in the mysteries and complexities of mechanosensory terminals.
